# A survey of *Fusobacterium nucleatum* genes modulated by host cell infection

**DOI:** 10.1099/mgen.0.000300

**Published:** 2019-10-29

**Authors:** Kyla Cochrane, Avery V. Robinson, Robert A. Holt, Emma Allen-Vercoe

**Affiliations:** ^1^​ Genome Sciences Center, BC Cancer Agency, Vancouver, British Columbia, V5Z 1L3, Canada; ^2^​ Molecular and Cellular Biology, University of Guelph, Guelph, Ontario, N1G 2W1, Canada; ^3^​ Molecular Biology and Biochemistry, Simon Fraser University, Burnaby, British Columbia, Canada; ^4^​ Medical Genetics, University of British Columbia, Vancouver, British Columbia, Canada; ^5^​ Canada's Michael Smith Genome Sciences Centre, Vancouver, British Columbia, Canada

**Keywords:** *Fusobacterium nucleatum*, transcriptome, infection

## Abstract

Here, we report comprehensive transcriptomic profiles from *Fusobacterium nucleatum* under conditions that mimic the first stages of bacterial infection in a highly differentiated adenocarcinoma epithelial cell line. Our transcriptomic *in vitro* adenocarcinoma approach allows us to measure the expression dynamics and regulation of bacterial virulence and response factors in real time, and is a novel strategy for clarifying the role of *F. nucleatum* infection in colorectal cancer (CRC) progression. Our data show that: (i) infection alters metabolic and functional pathways in *F. nucleatum *, allowing the bacterium to adapt to the host-imposed milieu; (ii) infection also stimulates the expression of genes required to help induce and promote a hypoxic and inflammatory microenvironment in the host; and (iii) *F. nucleatum* invasion occurs by a haematogenous route of infection. Our study identifies novel gene targets from *F. nucleatum* that are activated during invasion and which may aid in determining how this species invades and promotes disease within the human gastrointestinal tract. These invasion-specific genes may be useful as biomarkers for CRC progression in a host and could also assist in the development of new diagnostic tools and treatments (such as vaccines or small molecule drug targets), which will be able to combat infection and inflammation in the host while circumventing the potential problem of *F. nucleatum* tolerization.

## Data Summary

Raw data files have been deposited in the National Center for Biotechnology Information Gene Expression Omnibus under accession number GSE130714 (https://www.ncbi.nlm.nih.gov/geo/query/acc.cgi?acc=GSE130714).

**Impact Statement**

*
Fusobacterium nucleatum* is an emerging pathogen that has been implicated as a causal microbe in several diseases of the gastrointestinal tract, including Crohn’s disease and colorectal cancer. Although we know that this microbe can cause disease, how it does this remains enigmatic, partly because fusobacteria are difficult to culture and, therefore, study. In this work, we used a technique called RNA-seq (RNA sequencing) to understand the behaviour of the microbe as it interacts with host cells; this technique told us which genes *F. nucleatum* switches on in order to infect host cells and, therefore, offers a first glimpse of the molecular underpinnings of the process, as well as a potential list of targets that can be used to develop therapies to treat infections.

## Introduction

Adhesion and subsequent invasion of host cells by bacterial pathogens can initiate a dynamic cascade of events, resulting in altered gene expression in each of the organisms involved. Changes in gene expression patterns during infection can provide a global understanding of gene regulation, and reveal molecular detail concerning bacterial factors and pathways that underlie their ability to cause infections in the host [1]. These infectious processes are of particular concern in parts of the human body such as the colon, where microbes are present in high numbers and have constant contact with epithelial barriers [2, 3]. Evidence suggests that the microbial effects on host cells in the colon may contribute to the perpetuation of various inflammatory diseases, and can influence the development and progression of colorectal adenocarcinomas [4–6].

*Fusobacterium nucleatum* is well-recognized as a commensal bacterium on the mucosal surface, and extensive independent replication using metagenomic and transcriptomic analyses has shown *F. nucleatum* to be highly elevated in a subset of colorectal cancer (CRC) tissues [7–12]. In addition, *F. nucleatum* is an invasive bacterium that causes acute oral and gastrointestinal infections, and can act as a pro-inflammatory agent [13, 14]. Research has shown that in the presence of various *F. nucleatum* strains, colonic tumour formation in host cells is accelerated both *in vitro* and *in vivo* [15], anti-tumour immunity is suppressed [16], and tumour killing activity by natural killer cells is inhibited [17]; these findings collectively indicate a causative role for *F. nucleatum* in oncogenesis. However, not all *F. nucleatum* strains are associated with disease, which limits the diagnostic value of the species signature.

Using tissue culture models of bacterial invasion, we have previously shown that disease-associated *F. nucleatum* isolates tend to be more virulent than those recovered from healthy people [14]. It is likely that these disease-associated isolates possess virulence attributes that could be useful to distinguish them from benign isolates. Comparisons of sequenced genomes of *F. nucleatum* strains derived from both healthy and diseased patients have revealed high diversity and variability, with genes for many hypothetical proteins with no known homologies, and an apparent absence of ‘pathogenicity islands’ (hallmarks of bacterial pathogenesis) [18]. However, there is some evidence for the presence of virulence determinants in *F. nucleatum* and recent studies have begun to identify culprit adhesins/invasins that may be responsible, in part, for promoting *F. nucleatum* infection (e.g. *fadA* and *fap2*) [9, 10, 17].

Under the assumption that host cell invasion underscores *F. nucleatum* virulence – and that invasive *F. nucleatum* isolates have genes that are up-regulated to promote invasion – we employed a massively parallel, comprehensive and simultaneous whole-genome transcriptomic profiling approach (RNA-sequencing; RNA-seq), together with an established *in vitro* adenocarcinoma infection model, to assess *F. nucleatum* gene expression profiles during active invasion. The resulting gene expression profiles were validated using digital gene quantification and bar-coding software (NanoString). Subsequently, gene pathway analysis based on functional annotation of the differentially expressed genes was performed to highlight bacterial virulence pathways that were activated during infection.

## Methods

### *Fusobacterium* culture

Two clinical *F. nucleatum* isolates were used for this study: *F. nucleatum* subsp. *animalis* 7–1 (* F. nucleatum* 7-1) and *F. nucleatum* subsp. *animalis* 7–33 C1 (* F. nucleatum* 7–3). *F. nucleatum 7-1* has been previously isolated, extensively phenotyped and sequenced by our group [13, 14]. Although not isolated from a CRC biopsy, *F. nucleatum* 7–1 was chosen because it is associated with a finished genome [19] (to facilitate transcript mapping), has been extensively profiled in terms of adhesion and invasion assays [14], and has recently been shown to induce tumorigenesis in C57BL/6J–*Apc^Min/+^* mice [15]. *F. nucleatum* 7-3 was chosen because it was isolated directly from a CRC tumour biopsy through culture on selective medium [fastidious anaerobic agar (FAA) plates (Neogen) supplemented with 5 % (v/v) defibrinated sheep blood (Hemostat Laboratories) as well as josamycin (3 µg ml^−1^), vancomycin (4 µg ml^−1^) and norfloxacin (1 µg ml^−1^) (all antibiotics were from Sigma Aldrich)], has been extensively phenotyped and was highly invasive in our Caco-2 invasion model (data not shown). Propagation of *F. nucleatum* strains was carried out on FAA plates supplemented with 5 % (v/v) defibrinated sheep blood, or in tryptic soy broth supplemented with haemin (5 µg ml^−1^) and menadione (1 µg ml^−1^) (TSB_supp_) (Sigma-Aldrich), with incubation in a humidified anaerobic chamber (Ruskinn Bug Box) at 37 °C under an atmosphere of N_2_:CO_2_:H_2_ 90 : 5 : 5. For infection assays, *F. nucleatum* 7–1 and *F. nucleatum* 7–3 were grown in TSB_supp_ to late log phase and normalized for cell number (10^6^ c.f.u. ml^−1^), visually verified using McFarland standards.

### Caco-2 cell culture

Caco-2 human colon adenocarcinoma cells [American Type Culture Collection (ATCC) line HTB37] were used for *in vitro* infection assays. Cells were cultured in Dulbecco’s modified Eagle's medium (DMEM) (Sigma-Aldrich) supplemented with 10 % FBS (ThermoFisher), 10 mM sodium pyruvate (Sigma-Aldrich) and 5 µg Plasmocin ml^−1^ (Invitrogen) at 37 °C in 5 % CO_2_. For consistency, only Caco-2 cells from passages 4–12 were used for experiments.

### Experimental design, infection model and total RNA extraction

Caco-2 cells were grown to 85 % confluence, washed briefly with PBS and resuspended in supplemented DMEM without antibiotics. They were then infected at an m.o.i. of 100 : 1 with *F. nucleatum* 7–1 that had been grown to late log phase in TSB_supp_, and incubated at 37 °C in 5 % CO_2_ for 4 h [14]. Cells were then washed with sterile PBS and treated with fresh DMEM containing gentamicin (0.5 mg ml^−1^) (Sigma-Aldrich) for 30 min at 37 °C in 5 % CO_2_ to kill any bacteria present outside of the Caco-2 cells. The Caco-2 cells were then trypsinized and quenched. All subsequent steps were carried out using reagents at 4 °C. Cells were briefly pelleted by centrifugation before lysis with 0.1 % (v/v) Nonidet P-40 (Sigma-Aldrich), aided with gentle mixing to release internalized (invasive) *Fusobacterium* cells. Cells were once again pelleted by centrifugation and washed briefly with 1× Versene. RNA was stabilized through the addition of TRIzol reagent (1 ml per 50–100 mg pelleted sample) (ThermoFisher), before storage at −80 °C. RNAlater (Qiagen) was used in all buffers throughout the extraction process. Total RNA extraction was performed within 15 min to ensure minimal transcriptional changes during the process.

Total RNA was also isolated from TRIzol-stabilized samples of homogenized bacterial/tissue culture cells from the following control experiments: (i) *F. nucleatum* 7–1 grown in TSB_supp_; (ii) Caco-2 cells, with no bacterial exposure; and (iii) Caco-2 cells infected with *F. nucleatum* 7–1 cells for 4 h, collected after trypsinization but without gentamicin treatment. The parameters of control (iii) resolve *F. nucleatum* 7–1 genes that are needed for bacterial invasion into the host cell. The protocol for RNA isolation of all invasion and adhesion samples, and the respective controls were repeated with three biological replicates.

### RNA purification, bacterial ribodepletion and mRNA quantification

Samples were thawed, homogenized and RNA was separated from DNA and cellular debris by adding 0.2 ml chloroform per 1 ml TRIzol reagent followed by vigorous vortexing. Samples were then centrifuged at 12 000 ***g*** for 15 min at 4 °C, the aqueous phase was recovered and RNA was precipitated by adding 100 % isopropanol followed by centrifugation at 12 000 ***g*** for 10 min at 4 °C. The RNA pellet was washed three times in 75 % ethanol, air dried for 5 min and re-suspended in 50 µl nuclease-free, de-ionized H_2_O. Samples were treated with the Turbo DNA-free kit (ThermoFisher), the bacterial RNA fraction was enriched using the MICROB*enrich* kit (ThermoFisher), and the sample was then ribodepleted with the MICROB*express* kit (ThermoFisher), all following the manufacturer’s protocols. Finally, the quantity and quality of each RNA sample was assessed using Agilent Bioanalyzer 2100 RNA Nanochips.

### RNA-seq library preparation and sequencing

In total, 12 cDNA libraries (including biological replicates) containing both human and fusobacterial RNA were generated and sent for sequencing. The sequencing depth required for the accurate representation of both the bacterial pathogen and the mammalian host was set at a threshold of 200 million reads using estimates from previous studies [1, 20].

Library construction and Illumina sequencing were performed as described elsewhere [7, 21–23], but with the following modifications: (i) each paired-end library was PCR amplified for 15 cycles using the standard Illumina PE1 PCR primer; and (ii) a modified PE2 primer including a unique six base insertion as an index sequence was used. Libraries were gel-purified to remove residual adapter dimers and then sequenced on the Illumina HiSeq 2000 platform. Four paired-end 100 nt sequence lanes were run per library for a total of 12 lanes, yielding 952.6 million raw read pairs, well above the 200 million read threshold.

### *Fusobacterium* alignments and stranded gene coverage analysis

The Illumina paired-end sequence data were analysed with bwa alignment software to map each read pair onto the GCF_000158275.2_ASM15827v2 genomic reference fasta for the subspecies *F. nucleatum* 7–1, which was downloaded from the National Center for Biotechnology Information (NCBI) genome browser (https://www.ncbi.nlm.nih.gov) in April 2016.

bwa alignment version 0.5.7 was used to generate global alignments on the paired-end reads [24]. bwa was run with the default parameter settings used for alignments, except that the ‘-s’ option (to disable Smith–Waterman alignment) was used, because this feature was not designed to handle the insert size distribution that occurs in paired-end RNA-seq data. In the final stage of the initial alignment, all reads were excluded that failed Illumina's Chastity filter and turned on bit 512 in that record's bitwise flag to indicate the read failed platform/vendor quality checks.

For stranded gene coverage analysis, the annotations came from NCBI (GCF_000158275.2_ASM15827v2_genomic.*Fn*a) and correspond to the annotations in v30 of bacteria.ensembl.org. These annotations all include one annotation per gene and in this case NORM_TOTAL included all reads mapped to exons (since there was no mitochondrial sequence used for alignment). To determine each gene’s raw read count (each exons total unique reads), the reads per kilobase of transcript per million reads mapped (RPKM) strategy was used with the following formula [25]: (number of reads mapped to all exons in a gene × 1 000 000 000)/(NORM_TOTAL × sum of the lengths of all exons in the gene), where NORM_TOTAL=the total number of reads that are mapped to exons (i.e. fractional read count for exons).

### Differential gene expression and gene filtering

Differential gene expression statistical analysis between replicates was calculated using a two-tailed Student’s *t*-test with the [RPKM+1] values of bacterial controls versus the infection (adhesion and invasion) values. Genes and transcripts were defined as differentially expressed if they showed a log_2_ fold change ≥2.0 and a *P* value ≤0.05 (File S1, available with the online version of this article). This process served as a filtering step to select genes based on a nominal *P* value for further validation using the NanoString CodeSet.

### NanoString nCounter gene expression assay

Based on the RNA-seq differential gene expression results, specific probes for target sequences from the fusobacterial genes were designed and validated by NanoString to generate the Fuso CodeSet. A total of 26 bacterial (* F. nucleatum* 7–1) genes were selected and included both up- and down-regulated genes, as well as the housekeeping gene, *rpoB*. Genes were selected for inclusion in the NanoString CodeSet based on the combination of having an absolute fold-change value over 4 and a predicted annotated protein function related to invasion, pathogenesis or tumorigenesis. The complete list of genes and their respective probes for the Fuso CodeSet is provided in File S1.

The gene expression data was verified by NanoString nCounter technology, as described elsewhere [26]. Briefly, a total of 100 ng of each original RNA sample was hybridized to the Fuso CodeSet, which was composed of both capture and reporter probes. After incubation for 16 h at 37 °C, the samples were transferred to the nCounter Prep Station for binding and washing, and the cartridge was loaded onto the Digital Analyzer for transcript quantification. A total of 260 fields were captured per sample. The raw data were adjusted for binding efficiencies and background subtraction, following nCounter data analysis guidelines [26]. The data was then further normalized to the bacterial housekeeping gene *rpoB*. Gene expression ratios between RNA samples were calculated using mean values of three independent biological replicates, and statistical significance was determined using a two-tailed Student’s *t*-test (where significance required a *P* value ≥0.05). The coefficient of variation percentage was determined for each target gene based on the NanoString count data. If the variability across the three biological replicates was >0.6 (from two of the three controls, adhesion and/or invasion parameters) that gene was removed from the NanoString validated gene list altogether owing to a fault in the probe design.

To verify gene expression of the target genes during *F. nucleatum* infection, log2 ratios of gene expression levels were calculated from the NanoString count data to compare with the corresponding log2 ratio values from RNA-seq analysis using the Pearson’s correlation coefficient. Each gene was assessed individually and if the correlation between replicates was >0.5, the representative gene was considered unverified between the RNA-seq and NanoString data. All analyses for NanoString verification were performed using both the nSolver software and the R statistical software (version 3.3.0). All genes that passed the set thresholds from both RNA-seq and NanoString analyses were deemed activated or deactivated.

### Functional profiling for *Fusobacterium* gene expression

*F. nucleatum* genes activated during infection were included in KEGG pathway analysis using the KEGG Mapper Tool [27]. In addition, all activated *F. nucleatum* genes were annotated and the resulting amino acid sequence subjected to blastp searches against the Virulence Factor Database (VFDB) set A (core dataset) and set B (full dataset) [26, 28], which is a database of gene sequences representing all experimentally determined bacterial virulence factors.

### Validation of gene expression in an *in vitro* infection model with a CRC-derived *F. nucleatum* strain

To further validate gene expression during *Fusobacterium* invasion, an additional invasion assay was carried out using the CRC-derived isolate *F. nucleatum* 7–3. *F. nucleatum* 7–3 was used to infect Caco-2 cells and total RNA was extracted, purified and quantified on Agilent 2100 Bioanalyzer RNA Picochips, as described above. A total of 100 ng RNA from each sample was hybridized to the Fuso CodeSet for 16 h and subsequently transferred to the nCounter Prep Station and Digital Analyzer to determine relative expression levels of the target genes between control and invasion parameters. The fusobacterial housekeeping control gene, *rpoB*, was again used to normalize raw data for biological analyses [29]. Log_2_ ratios of gene expression levels were calculated to compare with the corresponding log_2_ ratio values from *F. nucleatum* 7–1 RNA-seq and NanoString data using the R statistical software package (version 3.3.0).

### Ethical approval

Strain *F. nucleatum* 7-3 was isolated from a biopsy obtained from the BC Cancer Agency Tumor Tissue Repository (research ethics approvals H09-01268 and H06-60001). Informed consent was provided from the donor for this purpose.

## Results

### Infection triggers pathogen transcriptional reprogramming

#### Fusobacterial transcription changes during adhesion and invasion

**Fig. 1.**
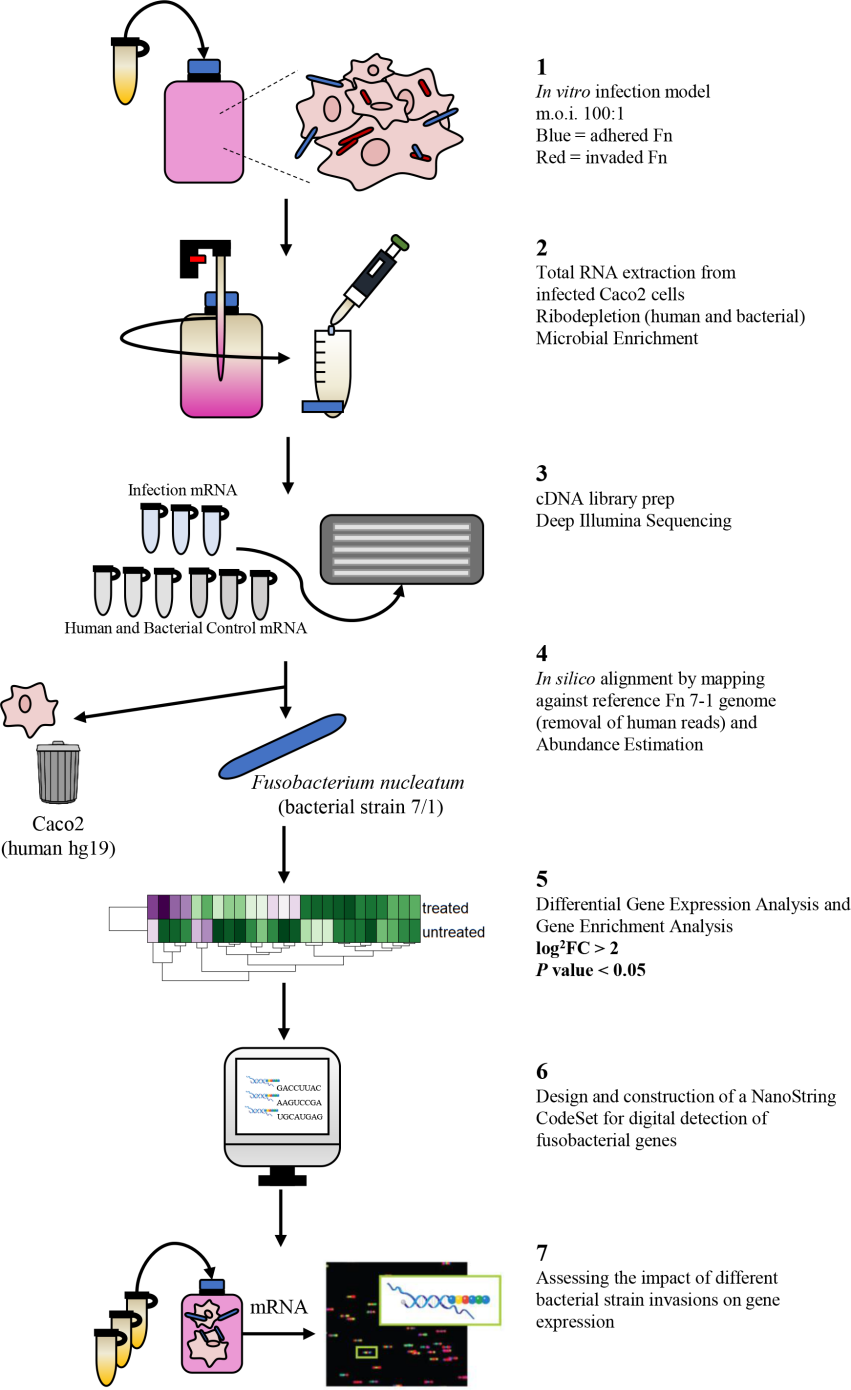
Experimental workflow for detecting differential expression using RNA-seq. Fn, *F. nucleatum *. FC: Fold Change

We dedicated much effort to performing our technically challenging infection model in a manner that was compatible with recovering RNA from invaded bacterial cells for RNA-seq, and to developing and applying a bioinformatics pipeline for analysing our results (Fig. 1).

Using R statistical software (version 3.3.0), the Spearman’s rank correlation coefficients for the monotonic relationship between RPKM-normalized values for the fusobacterial gene control, adhesion and invasion groups demonstrated >0.87, >0.94 and >0.96 correlations, respectively, indicating high reproducibility between replicates.

**Fig. 2.**
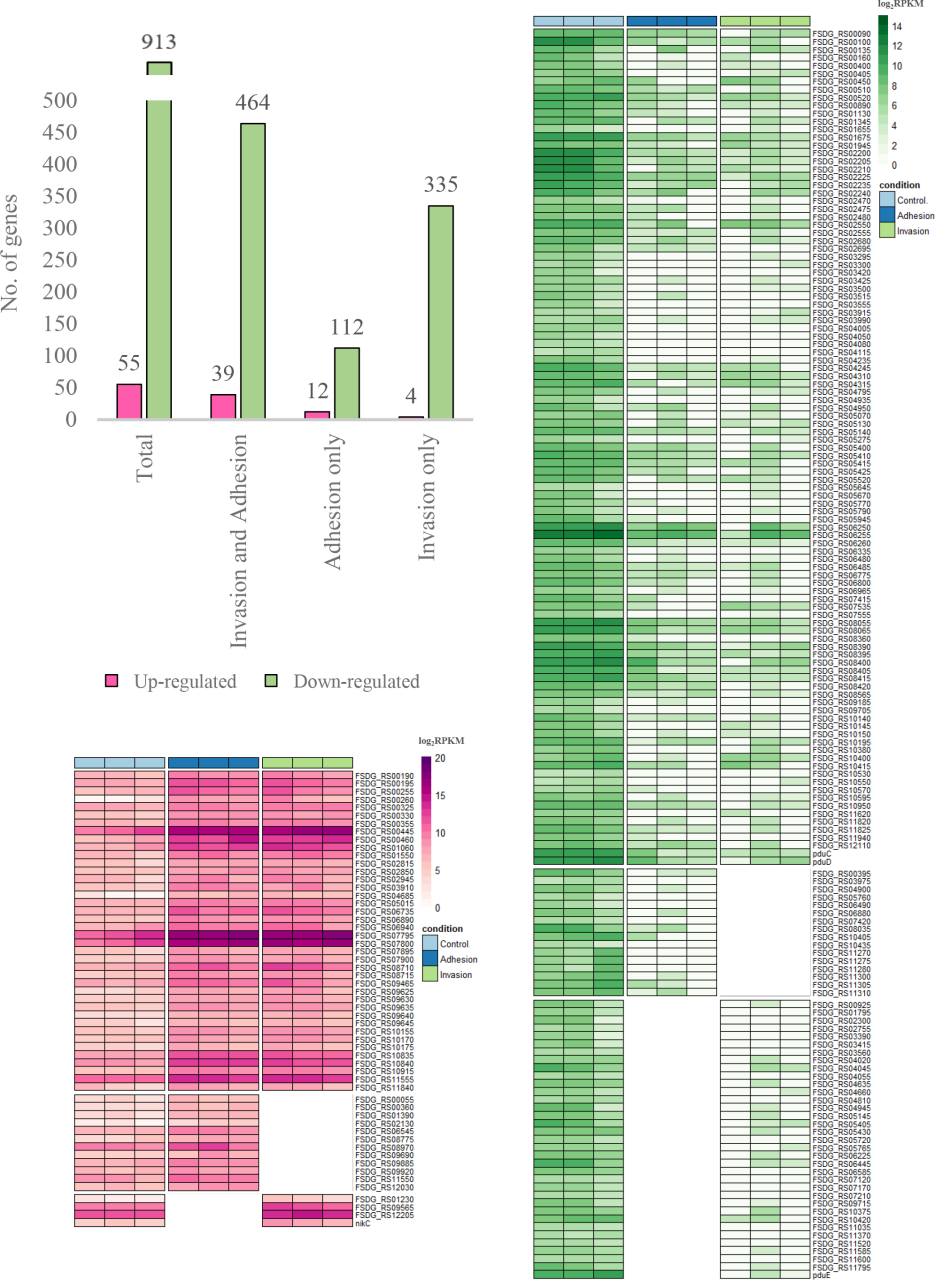
Heatmaps depicting the up-regulated and down-regulated genes as determined using the normalized RNA-seq data during both adhesion and invasion of *F. nucleatum* 7-1 in Caco2 cells. The bar graph represents a summary of the total number of genes found in each condition.

As expected, a low number of *Fusobacterium *-specific transcripts were found (a mean of 303 913 reads per replicate), which represent a small percentage of total mapped reads (0.6 %; File S1). Using a standard gene expression cut-off of RPKM≥0.1, a minimum of 10 mapped sequence reads and an absolute fold-change value of at least 4, we detected expression of 55 activated (and 913 deactivated) fusobacterial genes (Fig. 2, File S1).

**Fig. 3.**
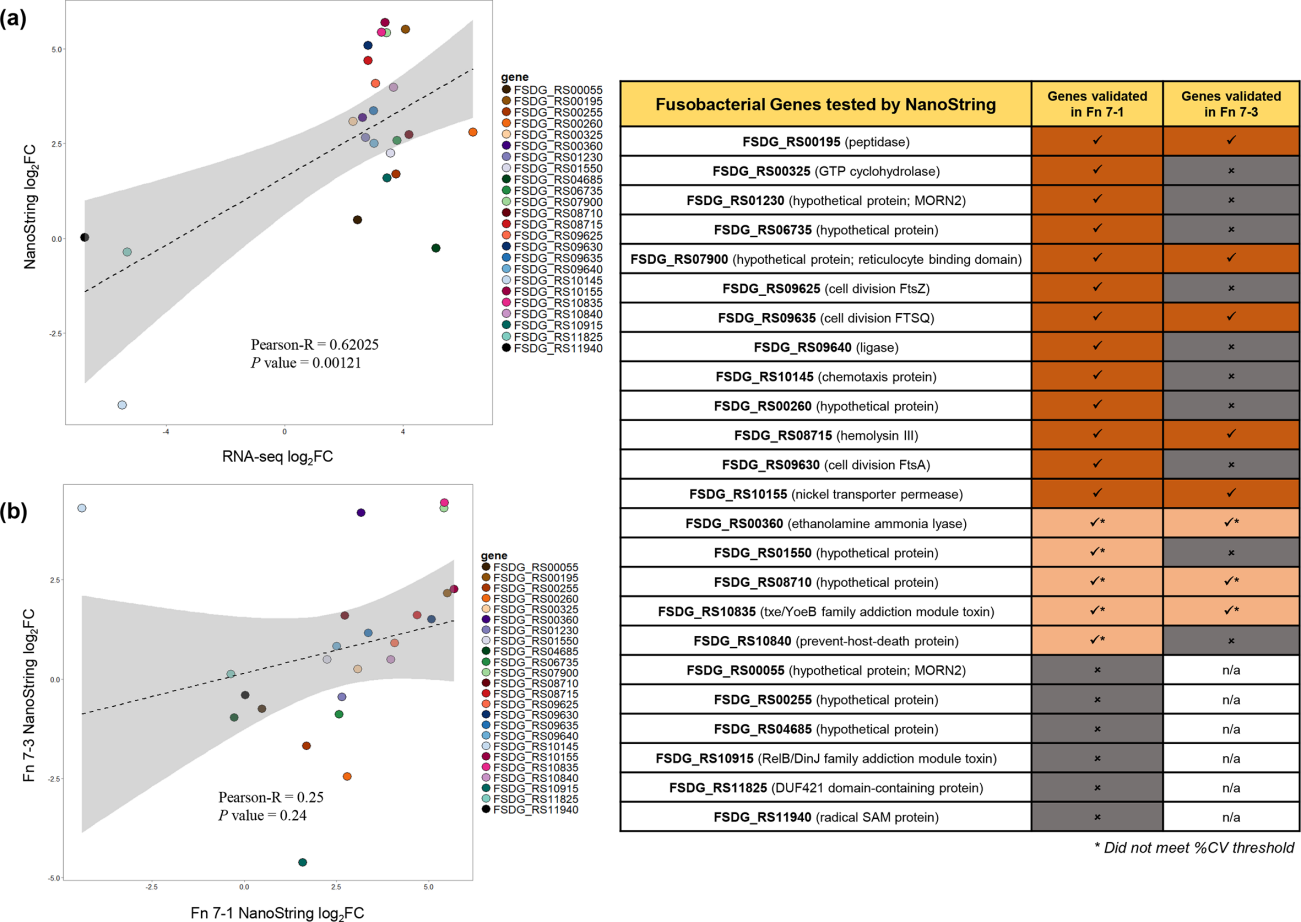
Correlation plots depicting the validation of *F. nucleatum* 7-1 gene up-regulation using the NanoString CodeSet and nCounter platform, and validation of the genes up-regulated in both *F. nucleatum* 7-1 and the CRC isolate *F. nucleatum* 7-3, as determined using the NanoString CodeSet. Fn, *F. nucleatum *. %CV: coefficient of variation; FC: Fold Change; n/a: not applicable.

Importantly, but also as expected, we observed that a significant portion of the highly up-regulated genes were annotated as having unknown functions and no clear cellular localization (for example no signal peptides, DNA binding motifs, etc.), which has led to their classification as hypothetical protein-encoding genes (15/55; 27.3%). Most of the activated genes were found in RNA extracted from both adherent and invasive bacteria (39/55; 70.9%). However, 4 unique genes were only significantly up-regulated during invasion (7.3%), and 12 unique genes were activated in bacteria that were preferentially bound to the surface of the Caco-2 cells but had not yet invaded (21.8%) (File S1). Following validation of gene expression using the NanoString platform, a total of 12 fusobacterial genes were confirmed to be up-regulated, and 1 gene was down-regulated, during the infection of Caco2 cells (Fig. 3; File S1).

#### Metabolic and functional pathways are modulated in *F. nucleatum* during infection

Functional analysis of the activated and verified genes from *F. nucleatum* was performed using kegg mapper pathway enrichment analysis software [27]. Host cell infection resulted in the up-regulation of metabolic pathways, quorum sensing, amino acid metabolism, protein export and the biosynthesis of folate, secondary metabolites, peptidoglycan and antibiotics (File S1). The up-regulation of many of these pathways is consistent with the idea that invasive *F. nucleatum* cells adapt to an intracellular lifestyle, and continue to replicate.

#### *F. nucleatum* modulates virulence factor expression during colonization

Bacterial virulence relies on a delicate balance of signals transmitted between the invading microbe and the host. This communication has been widely viewed as a battle involving harmful molecules produced by the pathogen and host defences. To detect the presence of known, well-documented virulence factors, we ran all infection-activated and validated *F. nucleatum* predicted amino acid sequences through the Virulence Factor Database (VFDB) via the blastp interface [26, 28]. A total of five genes came back with significant regions of homology, or hits, to known virulence protein domains with a bit score of >100 and *E* value >0.01 [FSDG_RS02815 (Lipopolysaccharide (LPS) biosynthesis protein); FSDG_RS08715 (haemolysin III); FSDG_RS09645 (UDP-*N*-acetylenolpyruvoylglucosamine reductase); FSDG_RS10170 (ATP binding protein); FSDG_RS10175 (ABC transporter ATP binding protein)] (File S1).

### Infection with CRC-associated strain *F. nucleatum* 7-3 results in similar activation of fusobacterial genes

Given the substantial genetic, antigenic and pathogenic diversity among *F. nucleatum* isolates [14, 18, 19], we acknowledge that differential gene expression may vary for *F. nucleatum* strains other than *F. nucleatum* 7–1 during infection in the host. To investigate this possibility, we used the custom NanoString CodeSet to look at the effect of strain variation on host gene expression. An additional invasive strain, *F. nucleatum* 7–3, was selected to detect gene expression changes between invasive *F. nucleatum* strains (File S1). We chose to take a conservative approach to analyses and report on only those genes that were up-regulated with a log fold change of at least 2 for both strains. A total of five fusobacterial genes were found to be up-regulated during invasion in both *F. nucleatum* 7–1 and *F. nucleatum* 7–3 [FSDG_RS00195 (peptidase); FSDG_RS07900 (hypothetical protein); FSDG_RS08715 (haemolysin III); FSDG_RS09635 (FtsQ); FSDG_RS10155 (nickel ABC transporter permease)].

## Discussion

*F. nucleatum* is an enigmatic species that is associated with a large number of human disease manifestations, and its most recent association with colon cancer reinforces the need for a better understanding of this pathogen. Although *F. nucleatum* is difficult to characterize – due partly to its high genomic heterogeneity and the lack of molecular mutagenesis tools – our research strategy combined transcriptomics with an *in vitro* infection model to create a suitable framework for assessing *F. nucleatum* gene expression during host cell infection. Here, we report comprehensive transcriptomic profiles from *F. nucleatum* to capture gene regulation during infection, using conditions that mimic the first stages of bacterial infection in a highly differentiated adenocarcinoma epithelial cell line.

### Transcriptome signatures highlight progression of infection and are consistent with a haematogenous route of infection

A preliminary finding from our RNA-seq assay results was that active infection with *F. nucleatum* 7–1 resulted in the up-regulation of two *F. nucleatum* genes – a membrane protein with a reticulocyte binding domain and haemolysin (FSDG_RS07900 and FSDG_RS08715). We corroborated this result using targeted digital detection by NanoString aimed at these same genes in our invasive CRC isolate, *F. nucleatum* 7–3. This is relevant because although *F. nucleatum* appears to be a natural inhabitant of the oral cavity, it is known to cause infections in a range of sites including the liver (e.g. pyogenous abscesses), the jugular vein (Lemierre’s disease) and the amniotic sac (leading to pre-term labour) [30–32]. It is thought that these infections are the result of haematogenous spread of *F. nucleatum* from the oral cavity [17, 33], and if this is the case, *F. nucleatum* haemolytic activity (as suggested by these two up-regulated genes) may be required.

### *F. nucleatum* infection enhances the host’s tumour environment by stimulating and promoting hypoxic and inflammatory conditions

Many factors are at play in the development of colon cancer, but microbes provide a major stimulus by developing and maintaining intestinal inflammation. Indeed, *F. nucleatum* infection induces extensive inflammation via activation of host pro-inflammatory mediators [13], such as oxygen radicals [34, 35], which through indirect host DNA damage may contribute to the CpG island methylation phenotype and microsatellite instability that have been seen in CRC tumour biopsy samples [10]. Micro-organisms have developed several mechanisms to survive in the host environment, such as competing with their host for metal acquisition and resisting host inflammatory defences such as reactive nitrogen species (RNS) and reactive oxygen species (ROS). Our analysis showed several *F. nucleatum* genes were active that may be related to the host’s release of ROS and RNS products: (i) GTP cyclohydrolase (FSDG_RS00325), a rate-limiting enzyme in the biopterin pathway known to prevent RNS uncoupling [36]; (ii) glutathione peroxidase (FSDG_RS09635), an enzyme capable of reducing free hydrogen peroxide to water [37] ; (iii) nickel transporter permease (FSDG_RS10155), an enzyme used in cells as part of hydrogenases or superoxide dismutases [38]. As a sulfate-reducing bacterial species, *F. nucleatum* can generate H_2_S, which scavenges O_2_ and thereby lowers the accumulation of ROS, removing free oxygen molecules from the tumour microenvironment [39]. Coincidentally, this may perpetuate a tumour phenomenon known as the Warburg effect, where cancer cells preferentially undergo glycolysis and enhanced glucose uptake followed by lactic acid fermentation, even in the presence of abundant oxygen, to produce the energy required for rapid cell growth [40]. Such metabolic changes in the host may be required to enable the synthesis of anti-microbial factors and pro-inflammatory mediators in direct response to *F. nucleatum* infection.

### *F. nucleatum* metabolic machinery adapts to the host milieu

*F. nucleatum* is an asaccharolytic bacterium and so will not compete for glucose. Instead, in the anaerobic microenvironment of the developing tumour, *F. nucleatum* utilizes amino acids and peptides as nutrients. Many of the by-products of amino acid metabolism, including short-chain fatty acids, may make the tumour microenvironment more tumour-permissive over time by directly promoting tumour cell proliferation, blood vessel growth or immune cell infiltration [10]. In support of this theory, we noted an up-regulation in the gene encoding the metabolic enzyme GTP cyclohydrolase (FSDG_RS00325). As well as its activity in protecting against oxidative damage (see above), this enzyme converts GTP to dihydroneopterin triphosphate in the folate pathway to produce folic acid, which has been linked to cancer progression due to its ability to promote cellular replication in the host [41]. There is also evidence that *F. nucleatum* invasion into the host cell does not disrupt the bacteria’s ability to replicate, as proteins involved in cell division (FSDG_RS9625, FSDG_RS09630, FSDG_RS09635) and peptidoglycan synthesis (FSDG_RS9640) are up-regulated. Indeed, since stationary phase cultures were added to host cells at the start of the invasion assay, up-regulation of cell division and peptidoglycan synthesis genes points to the ability for the intracellular fusobacterial cells to adapt and persist within the host cell environment, despite their strictly anaerobic physiology. In our hands, viable intracellular *F. nucleatum* strains can be recovered from Caco-2 cells up to 48 h post-inoculation (unpublished observations). Recent data suggests that, *in vivo*, *F. nucleatum* may persist for some time inside host cells, and mouse xenografts of primary colorectal adenocarcinomas could support viable *F. nucleatum* through several passages [42].

### Detection of invasion/adhesion-associated genes

One goal of this project was to identify protein-encoding genes that suggest alternative mechanisms for fusobacterial adhesion and invasion into host epithelial cells. We also wanted to assess the two most thoroughly described fusobacterial adhesins/invasins (namely *fadA* and *fap2*), using our infection model. The novel *Fusobacterium* adhesin, FadA, is associated with attachment and invasion of host cells through the binding of cadherins [both vascular endothelial (VE) and epithelial (E) types] and the consequent activation of host Wnt/β-catenin signalling pathways. These events can lead to bacterial internalization as well as the induction of both oncogenic and inflammatory responses in the host [11, 16, 43]. However, FadA is ubiquitously expressed in *F. nucleatum* strains, and it is highly expressed in even weakly invasive isolates [18]. Furthermore, while VE/E-cadherin may support *F. nucleatum* attachment/invasion, it cannot be the sole factor mediating host cell invasion, because it is expressed by many host cell types and its cellular localization patterns in dysplasia and neoplasia vary substantially [44, 45]. Other work has shown that the *F. nucleatum* lectin Fap2 recognizes host Gal-GalNAc (a polysaccharide over-expressed in CRC) [45]. Fap2-dependent bacterial attachment occurs under the constant flow conditions of the oral cavity where surface adhesion is vital, but this signal may be absent in the adenocarcinoma niche. In addition, although *F. nucleatum* binding to adenocarcinomas correlates with Gal-GalNAc expression, *F. nucleatum* strains lacking Fap2, including CRC-derived isolates, can still exhibit marked interaction with CRC cells [46]. Overall, it appears that Fap2 may aid in the haematogenous spread and fusobacterial enrichment in CRC in human tissues, but does not appear to be responsible for the subsequent colonic invasion of the bacterial species.

Interestingly, neither *fadA* nor *fap2* were found to be significantly modulated in the current study, which highlights the importance of a comprehensive view of microbe/host interaction such as that presented here. Our study has identified 15 hypothetical proteins that could be involved in the invasion of *F. nucleatum* into host cells. FSDG_RS01230 is a particularly strong candidate for additional study as it contains MORN2 domains, which have been predicted to play a role in adhesion and active invasion [18]. MORN2 domain containing proteins are encoded in many *F. nucleatum* strains within sets of genes with no known function, that tend to be clustered in the same genomic neighbourhoods as other adhesins, including *fadA*. MORN2 domains are common in invasive fusobacterial species and have been predicted to function to advance adhesive, aggregative and invasive traits within fusobacterial species [18]. Our study provides further weight to this finding and suggests that at least 1 MORN2 domain containing gene product could be an attractive target for therapeutic intervention.

### Conclusion

Our transcriptomic *in vitro* adenocarcinoma approach allows us to measure the expression dynamics of virulence and response factors in real time, and is a novel strategy for clarifying the role of *F. nucleatum* infection in CRC progression. Specific genes have been identified as potential targets for future mechanistic studies into *F. nucleatum* invasion within the human gastrointestinal tract. This new information increases our knowledge of this bacterium and its interaction with host cells, and may assist in the development of new diagnostic tools, and ultimately new treatments (such as vaccines or small molecule drug targets), which will be able to combat infection and inflammation in the host while circumventing the potential problem of *F. nucleatum* tolerization.

## Data bibliography

1. K. C. The Illumina paired-end sequence data were analysed with bwa alignment software to map each read pair onto the GCF_000158275.2_ASM15827v2 genomic reference fasta for the subspecies *F. nucleatum* 7-1, which was downloaded from the NCBI genome browser (www.ncbi.nlm.nih.gov) in April 2016.

2. K. C. For stranded gene coverage analysis, the annotations came from NCBI (GCF_000158275.2_ASM15827v2_genomic.Fna) and correspond to the annotations in v30 of bacteria.ensembl.org. (2016).

## Supplementary Data

Supplementary material 1
